# Feasibility and effectiveness of trifluridine/tipiracil in metastatic colorectal cancer: real-life data from The Netherlands

**DOI:** 10.1007/s10147-017-1220-0

**Published:** 2017-12-04

**Authors:** Johannes J. M. Kwakman, G. Vink, J. H. Vestjens, L. V. Beerepoot, J. W. de Groot, R. L. Jansen, F. L. Opdam, H. Boot, G. J. Creemers, J. M. van Rooijen, M. Los, A. J. E. Vulink, H. Schut, E. van Meerten, A. Baars, P. Hamberg, E. Kapiteijn, D. W. Sommeijer, C. J. A. Punt, M. Koopman

**Affiliations:** 1 Department of Medical Oncology, Academic Medical Center, University of Amsterdam, Room F4-224, Meibergdreef 9, 1105 AZ Amsterdam, The Netherlands; 2 Netherlands Comprehensive Cancer Organisation (IKNL), Godebaldkwartier 419, 3511 DT Utrecht, The Netherlands; 3 Department of Internal Medicine, Viecuri Hospital, Tegelseweg 210, 5912 BL Venlo, The Netherlands; 4 Department of Medical Oncology, Elisabeth-TweeSteden Hospital, Doctor Deelenlaan 5, 5042 AD Tilburg, The Netherlands; 5 Department of Medical Oncology, Isala Clinics, Dokter van Heesweg 2, 8025 AB Zwolle, The Netherlands; 6 Department of Medical Oncology, Maastricht University Medical Center, P. Debyelaan 25, 6229 HX Maastricht, The Netherlands; 7 Department of Medical Oncology, The Netherlands Cancer Institute, Plesmanlaan 121, 1066 CX Amsterdam, The Netherlands; 8 Department of Gastroenterology and Hepatology, The Netherlands Cancer Institute, Plesmanlaan 121, 1066 CX Amsterdam, The Netherlands; 9 Department of Medical Oncology, Catharina Hospital, Michelangelolaan 2, 5623 EJ Eindhoven, The Netherlands; 10 Department of Medical Oncology, Martini Hospital, Van Swietenplein 1, 9728 NT Groningen, The Netherlands; 11 Department of Medical Oncology, St. Antonius Hospital, Koekoekslaan 1, 3435 CM Nieuwegein, The Netherlands; 12 Department of Medical Oncology, Reinier de Graaf Gasthuis, Reinier de Graafweg 5, 2625 AD Delft, The Netherlands; 13 Department of Medical Oncology, Jeroen Bosch Hospital, Henri Dunantstraat 1, 5223 GZ Den Bosch, The Netherlands; 14 Department of Medical Oncology, Erasmus Medical Center, Erasmus University, ‘s-Gravendijkwal 230, 3015 CE Rotterdam, The Netherlands; 15 Department of Medical Oncology, Hospital Gelderse Vallei Ede, Willy Brandtlaan 10, 6716 RP Ede, The Netherlands; 16 Department of Medical Oncology, Franciscus Gasthuis, Kleiweg 500, 3045 PM Rotterdam, The Netherlands; 17 Department of Medical Oncology, Leiden University Medical Center, Albinusdreef 2, 2333 ZA Leiden, The Netherlands; 18 Department of Medical Oncology, Flevo Hospital, Hospitaalweg 1, 1315 RA Almere, The Netherlands; 19 Department of Medical Oncology, University Medical Center Utrecht, Heidelberglaan 100, 3584 CX Utrecht, The Netherlands; 20 Academic Medical Center, University of Amsterdam, P.O. Box 22660, 1100 DD Amsterdam, The Netherlands

**Keywords:** Trifluridine/tipiracil, TAS-102, Metastatic colorectal cancer, Compassionate use, Feasibility

## Abstract

**Background:**

The RECOURSE trial showed clinical efficacy for trifluridine/tipiracil for refractory metastatic colorectal cancer patients. We assessed the feasibility and effectiveness of trifluridine/tipiracil in daily clinical practice in The Netherlands.

**Methods:**

Medical records of patients from 17 centers treated in the trifluridine/tipiracil compassionate use program were reviewed and checked for RECOURSE eligibility criteria. Baseline characteristics, safety, and survival times were compared, and prespecified baseline characteristics were tested in multivariate analyses for prognostic significance on overall survival (OS).

**Results:**

A total of 136 patients with a median age of 62 years were analyzed. Forty-three patients (32%) did not meet the RECOURSE eligibility criteria for not having received all prior standard treatments (*n* = 35, 26%) and/or ECOG performance status (PS) 2 (*n* = 12, 9%). The most common grade ≥3 toxicities were neutropenia (*n* = 44, 32%), leukopenia (*n* = 8, 6%), anemia (*n* = 7, 5%), and fatigue (*n* = 7, 5%). Median progression-free survival (PFS) and median OS were 2.1 (95% CI, 1.8–2.3) and 5.4 months (95% CI, 4.0–6.9), respectively. Patients with ECOG PS 2 had a worse median OS (3.2 months) compared to patients with ECOG PS 0–1 (5.9 months). ECOG PS, *KRAS*-mutation status, white blood cell count, serum lactate dehydrogenase, and alkaline phosphatase were prognostic factors for OS.

**Conclusions:**

Our data show that treatment with trifluridine/tipiracil in daily clinical practice is feasible and safe. Differences in patient characteristics between our population and the RECOURSE study population should be taken into account in the interpretation of survival data. Our results argue against the use of trifluridine/tipiracil in patients with ECOG PS 2.

**Funding:**

Johannes J.M. Kwakman received an unrestricted research grant from Servier.

## Introduction

Recently, trifluridine/tipiracil (TAS-102) has shown efficacy in refractory metastatic colorectal cancer (mCRC). The randomized phase 3 RECOURSE trial showed a significant increase in median overall survival (OS) of 1.8 months compared to placebo in refractory mCRC patients [1]. Trifluridine is a thymidine analogue that is incorporated into DNA, causing its antitumor effect [2]. Tipiracil hydrochloride prevents the rapid degradation of trifluridine, which allows for continuous adequate plasma levels of trifluridine [3, 4].

Clinical trial results provide the backbone of evidence-based medicine and are incorporated in clinical guidelines. However, a trial population may not always be a representative sample of the total population because of restrictive trial eligibility criteria. For example, in the RECOURSE study only patients with an Eastern Cooperative Oncology Group performance status (ECOG PS) of 0 or 1 were included. Also, the controlled conditions under which clinical trials are usually performed cannot always be guaranteed in general practice. Hence, the use of observational data of non-trial patients may be helpful to assess the feasibility and effectiveness of novel treatments in daily clinical practice.

After publication of the results of RECOURSE [1], a compassionate use program for trifluridine/tipiracil in refractory mCRC patients was initiated in The Netherlands before market access January 2017. We analyzed the baseline characteristics of these patients with the RECOURSE eligibility criteria and assessed the feasibility and effectiveness of trifluridine/tipiracil treatment in this non-trial population. Finally, these data allowed us to evaluate patient and tumor characteristics for prognostic significance on OS.

## Methods

### Patients

Physicians who registered patients between December 2015 and January 2017 for the trifluridine/tipiracil compassionate use program in The Netherlands were invited to participate in the study. Participation criteria for the compassionate use program were comparable but not equal to the eligibility criteria of the RECOURSE study and included a biopsy-confirmed adenocarcinoma of the colon or rectum with the presence of metastatic lesions. Patients were required to have received at least two prior regimens of standard chemotherapies, which may have included adjuvant chemotherapy if a tumor had recurred within 6 months, and exposure to a fluoropyrimidine, oxaliplatin, irinotecan, bevacizumab, and, for patients with *(K)RAS*-wild-type tumors, an anti-epidermal growth factor receptor (EGFR) monoclonal antibody. Patients must have been refractory to those therapies or significant adverse events must have precluded their readministration. In addition, patients must have adequate bone marrow, liver, and renal function. Medical records of patients were reviewed, and baseline clinicopathological factors including (but not limited to) sex, age, disease characteristics, prior systemic regimens, (*K)RAS*-mutation status, ECOG PS, serum lactate dehydrogenase (LDH), alkaline phosphatase (ALP), white blood cell count (WBC), number of trifluridine/tipiracil cycles, toxicities, number of dose delays and dose reductions, disease response, date of progression, and date of death were collected. All patients provided written informed consent for the treatment. The study was reviewed by the Medical Ethics Review Committee of the Academic Medical Center, Amsterdam, who decided that the Medical Research Involving Human Subjects Act (WMO) did not apply to the study.

### Treatment

According to the RECOURSE study, the recommended dose of trifluridine/tipiracil treatment was 35 mg/m^2^ twice daily, for 5 days per week with 2 days of rest for 2 weeks, followed by 2 weeks of rest. This schedule was repeated every 4 weeks until disease progression, unacceptable toxicity, or patient refusal occurred.

### Outcomes

Patient baseline characteristics were checked for RECOURSE eligibility and compared to the RECOURSE study population. Study endpoints included safety, median OS, median progression-free survival (PFS), and disease control rate (DCR). The survival times and DCR of eligible and ineligible patients were compared. Last, prespecified variables were evaluated for prognostic significance on OS. All toxicities were scored according to National Cancer Institute Common Terminology Criteria for Adverse Events (NCI CTCAE), version 4.0. Tumor response was evaluated according to RECIST 1.1 at intervals at the discretion of the treating physicians.

### Statistical analysis

Patients who received at least one dose of trifluridine/tipiracil were included in the analysis. Descriptive statistics and frequency tables were used to characterize the study population. PFS was defined as the time from trifluridine/tipiracil initiation to the date of first documented progression or death from any cause. OS was defined as the time from treatment initiation to the date of death from any cause. Median PFS and OS were estimated with the Kaplan–Meier method and compared with Cox proportional hazard models. Patients alive or alive without progression at last follow-up were censored for OS and PFS, respectively. The response rate (RR) represents the proportion of patients with complete or partial response. DCR represents the proportion of patients with complete response, partial response, or stable disease, and was compared with Fisher’s exact test.

Prespecified variables for the identification of potential prognostic factors on OS were categorized based on clinical reasoning and taking subgroup sizes into account as follows: sex (male versus female), age (<70 years versus ≥70 years), resection of primary tumor (yes versus no), tumor sidedness (right colon versus left colon and rectum with splenic flexure as differentiation), number of metastatic sites (1 versus 2 and ≥3), presence of peritoneal metastases (yes versus no), exposure to all standard treatments (yes versus no), time from diagnosis of metastases and start treatment (<18 months versus ≥18 months), ECOG PS (0 versus 1 or 2), LDH (<350 versus ≥350 U/l), WBC (<8.0 versus ≥8.0 × 10^9^/l), ALP (<200 versus ≥200 U/l), and (*K)RAS*-mutation status (mutated versus wild type). Multivariate associations between these variables and OS were examined using a Cox regression model from which hazard ratios (HR) were obtained with 95% confidence intervals (CI). *p* values less than 0.05 were considered to indicate statistical significance.

## Results

### Study population

A total of 148 registered patients from 17 centers were reviewed. Trifluridine/tipiracil treatment was started in 136 patients, who were further analyzed. Twelve patients (9%) did not fulfill the RECOURSE study eligibility criteria because of an ECOG PS of 2 at the time of treatment initiation. Eight patients (6%) had not received at least two prior regimens. Of these patients, 4 were considered unfit for treatment with second-line oxaliplatin or irinotecan, 3 had had significant toxicity upon adjuvant chemotherapy (>6 months before to metastatic disease) that precluded the readministration of a fluoropyrimidine (*n* = 2) or oxaliplatin (*n* = 1), and 1 was treated with a combination of a fluoropyrimidine, oxaliplatin, irinotecan, and bevacizumab as the only prior regimen. An additional 35 patients (42 in total, 31%) did not receive all standard treatments, of which 5 patients were not retreated with oxaliplatin because of significant toxicities in the adjuvant setting, 3 patients refused irinotecan treatment, and 3 and 1 patients were considered unfit for oxaliplatin and irinotecan, respectively. A total of 37 patients (27%) were not pretreated with bevacizumab, 30 for unknown reasons and 7 for medical considerations. All patients with a (*K)RAS*-wild-type tumor had received anti-EGFR monoclonal antibodies. A total of 43 patients (32%) were considered to be ineligible according to the RECOURSE eligibility criteria. Compared to the RECOURSE study population, more patients were male (68% versus 61%), more patients had ECOG PS 1 (57% versus 44%) or 2 (9% versus 0%), fewer patients had received all standard treatments (69% versus 100%), or had been refractory to fluoropyrimidines (90% versus 98%), or had a (*K)RAS*-wild-type tumor (39% versus 49%). The main baseline characteristics of RECOURSE study patients and our non-trial population, with differentiation between ineligible and eligible patients, are listed in Table 1.

**Table 1:** Baseline characteristics

	Recourse	Compassionate use program		
	Trifluridine/tipiracil group	All patients	Eligible patients	Ineligible patients
	*n* = 534 (%)	*n* = 136 (%)	*n* = 93 (%)	*n* = 43 (%)
Sex				
Male	326 (61)	92 (68)	63 (68)	29 (67)
Female	208 (39)	44 (32)	30 (32)	14 (33)
Age (years, median), range	63 (27–82)	62 (30–88)	61 (30–81)	65 (34–88)
ECOG performance status				
0	301 (56)	46 (34)	33 (36)	12 (28)
1	233 (44)	78 (57)	60 (65)	19 (44)
2	0 (0)	12 (9)	0 (0)	12 (28)
*KRAS*-mutation				
No	262 (49)	53 (39)	33 (36)	20 (47)
Yes	272 (51)	83 (61)	60 (65)	23 (54)
Time from diagnosis of metastases				
<18 months	111 (21)	29 (21)	18 (19)	11 (26)
≥18 months	423 (79)	107 (79)	75 (81)	32 (74)
Number of prior regimens				
1	0 (0)	8 (6)	3 (3)	5 (12)
2	95 (18)	54 (40)	36 (39)	18 (42)
3	119 (22)	56 (41)	41 (44)	15 (35)
≥4	320 (60)	18 (13)	13 (14)	5 (12)
Prior systemic anticancer agents		^a^	^a^	^a^
Fluoropyrimidine	534 (100)	134 (99)	91 (98)	43 (100)
Oxaliplatin	534 (100)	121 (89)	90 (97)	31 (72)
Irinotecan	534 (100)	128 (94)	93 (100)	35 (81)
Bevacizumab	534 (100)	99 (73)	86 (93)	12 (28)
Anti-EGFR monoclonal antibodies	278 (52)	53 (39)	33 (36)	20 (47)
Refractory to fluoropyrimidine	524 (98)	123 (90)	84 (90)	39 (91)

*ECOG* Eastern Cooperative Oncology Group

^a^ In metastatic setting or as adjuvant treatment <6 months before recurrent disease

### Safety

All patients started treatment at the recommended dose. The median number of cycles was 2 (range, 1–10). At the time of the analysis, 123 patients (90%) had discontinued treatment of which 5 (4%) resulted from treatment-related toxicities. Five patients (4%) were hospitalized because of treatment-related adverse events, which included febrile neutropenia in 3 patients, and 1 patient each for ileitis and a combination of mucositis, nausea, and vomiting, respectively. There were no treatment-related deaths. A total of 38 dose reductions were applied in 31 patients (23%), with 26 (19%), 3 (2%), and 2 (1%) patients having 1, 2, or 3 reductions, respectively. Dose delays were applied in 53 patients (39%; range, 0–7). The most common grade ≥3 adverse event was neutropenia (*n* = 44, 32%). Grade ≥3 leukopenia, anemia, and fatigue were reported in 8 (6%), 7 (5%), and 7 (5%) patients, respectively. Other commonly observed toxicities included nausea, anorexia, and diarrhea, but these events were mostly limited to grade 1 and 2 (Table 2).

**Table 2:** Treatment-related adverse events (AEs)

	*n* = 136 (%)
Any AEs	103 (76)
Grade ≥3 AEs	60 (44)
Nonhematological grade ≥3 AEs	16 (12)
Most common AEs, any grade	
Diarrhea	16 (12)
Nausea	26 (19)
Vomiting	7 (5)
Anorexia	21 (15)
Mucositis	9 (7)
Fatigue	50 (37)
Grade ≥3 laboratory abnormalities	
Neutropenia	44 (32)
Leukopenia	8 (6)
Anemia	7 (5)
Thrombocytopenia	1 (1)
Increased total bilirubin	2 (2)
Serious AEs	5 (4)
Resulting from febrile neutropenia	3 (2)
Resulting from gastrointestinal toxicities	2 (2)

*AE* adverse event

Compared to the RECOURSE population, fewer grade ≥3 events (69% versus 44%) and serious events (30% versus 4%) were reported, whereas dose reductions were applied in a greater proportion of patients (14% versus 23%). A comparable number of patients discontinued treatment because of adverse events (4% versus 4%).

### Efficacy

**Fig. 1:**
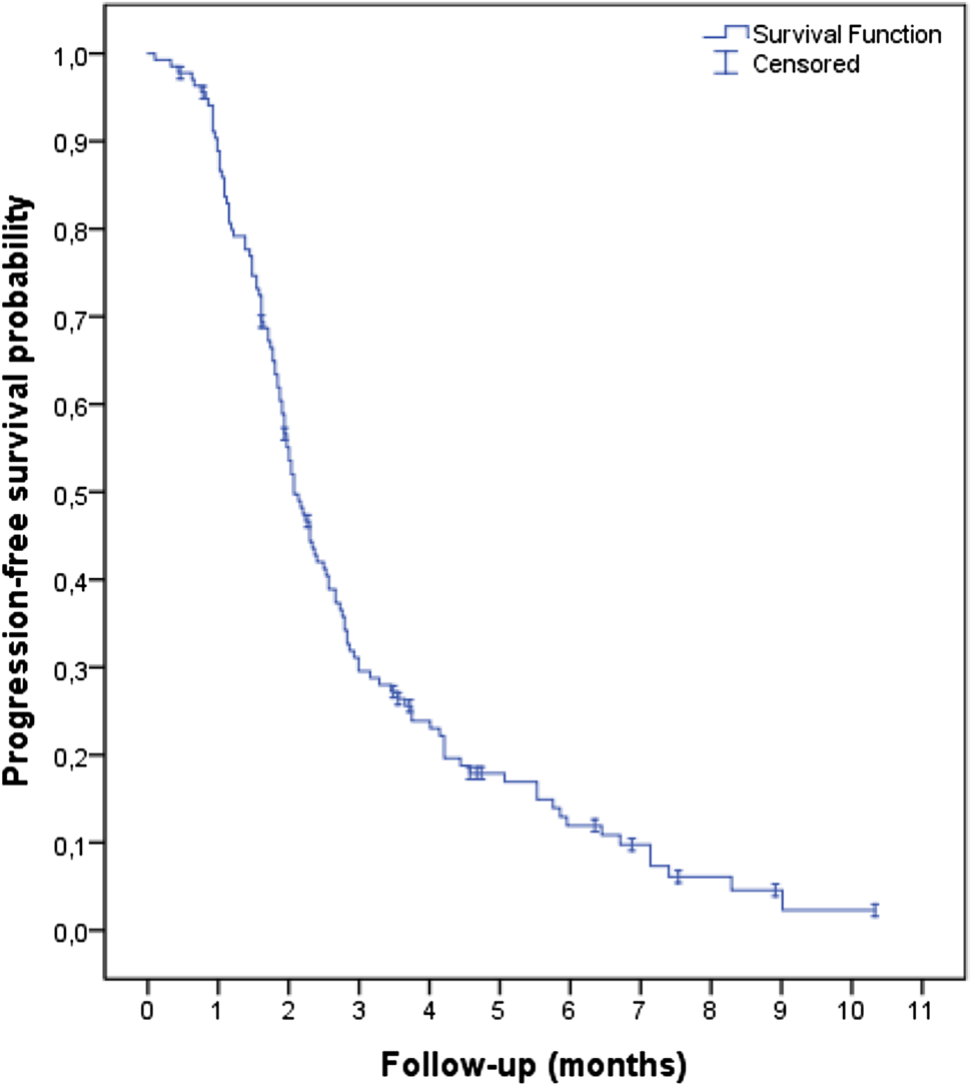
Kaplan–Meier curve of progression-free survival

**Fig. 2:**
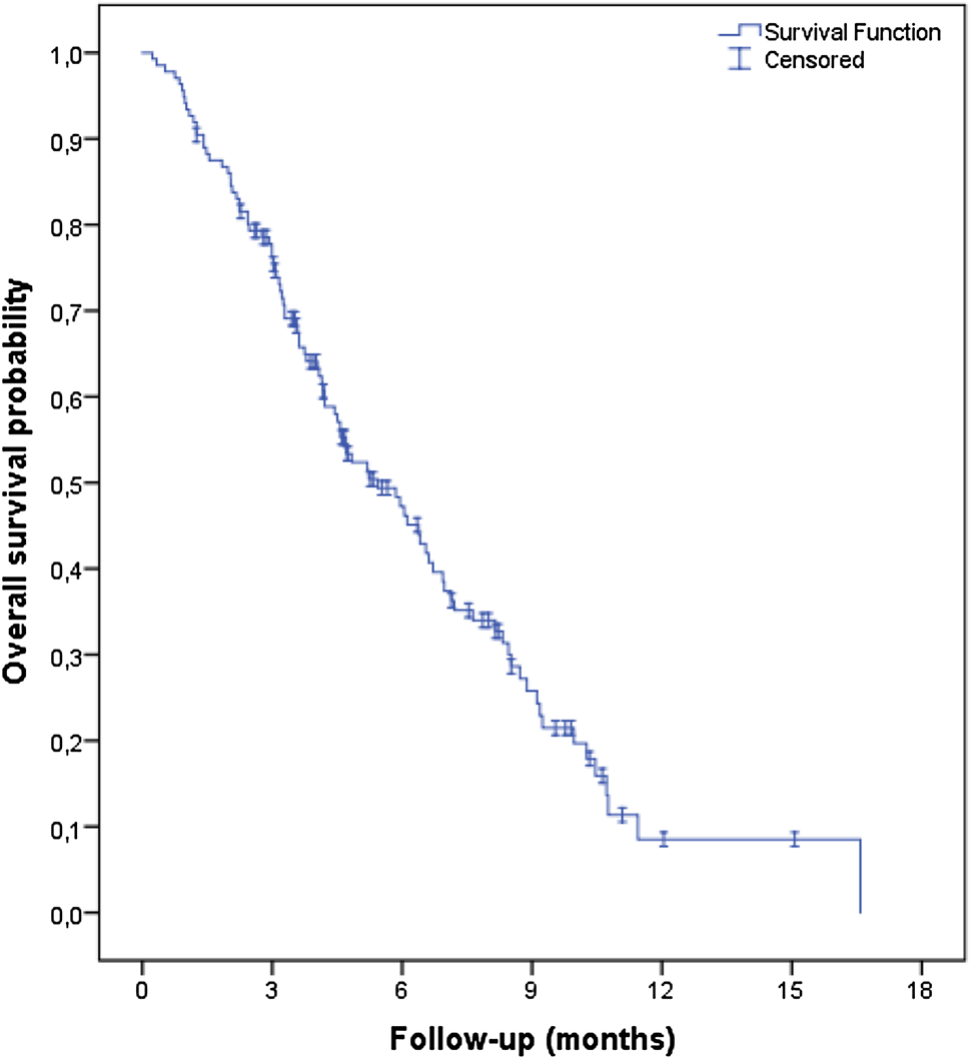
Kaplan–Meier curve of overall survival

After a median follow-up of 4.2 months, 120 patients (88%) had progressed and 93 patients had died (68%). Median PFS was 2.1 months (95% CI, 1.8–2.3; Fig. 1), and median OS was 5.4 months (95% CI, 4.0–6.9; Fig. 2). A total of 132 patients were evaluated for response. Two patients had a partial response and 35 patients had stable disease, resulting in a RR of 2% and DCR of 28%.

These outcomes are worse when compared to the outcomes of the RECOURSE study population (Table 3). Patients who were ineligible according to the RECOURSE study criteria had superior efficacy results compared to eligible patients, with a DCR of 48% versus 20% (*p* = 0.002), median PFS of 2.8 months (95% CI, 1.2–4.4) versus 2.0 months (1.9–2.2; HR, 0.53, 95% CI, 0.35–0.81; *p* = 0.003) and median OS of 8.2 months (5.0–11.4) versus 4.8 months (3.6–6.0; HR, 0.61, 95% CI, 0.38–0.95; *p* = 0.04). Subgroup analyses indicated that the subgroup of patients who did not receive all standard therapies before trifluridine/tipiracil (for reasons not meeting the RECOURSE eligibility criteria, *n* = 35) had a superior median OS (8.5 months; 95% CI, 5.2–11.8) as compared to patients who were fully pretreated [or significant adverse events precluded the readministration of any of those therapies; *n* = 101; median OS, 4.7 months (3.6–5.8); HR, 0.51, 95% CI, 0.30–0.85; *p* = 0.01]. Patients with ECOG PS 0–1 (*n* = 124) had a median OS of 5.9 months (95% CI, 4.4–7.3), compared to 3.2 months (1.4–5.0) for patients with ECOG PS 2 (*n* = 12; HR, 0.79, 95% CI, 0.36–1.71; *p* = 0.54). Median OS was 4.6 months (95% CI, 4.0–5.1) and 6.9 months (4.2–9.7) for patients with a *KRAS*-mutated tumor (*n* = 83) and *KRAS*-wild-type tumor (*n* = 53), respectively (HR, 1.67, 95% CI, 1.08–2.59; *p* = 0.02).

**Table 3:** Treatment outcomes

	Recourse	Compassionate use program		
	Trifluridine/tipiracil group	All patients	Eligible patients	Ineligible patients
	*n* = 534 (%)	*n* = 136 (%)	*n* = 93 (%)	*n* = 43 (%)
Overall response^a^	6 (1.6)	2 (2)	0 (0)	2 (5)
Disease control^a^	221 (44)	37 (28)	18 (20)	19 (48)
Progression-free survival (months, median), 95% CI	2.0 (1.9–2.1)	2.1 (1.8–2.3)	2.0 (1.9–2.2)	2.8 (1.2–4.4)
Overall survival (months, median), 95% CI	7.1 (6.5–7.8)	5.4 (4.0–6.9)	4.8 (3.6–6.0)	8.2 (5.0–11.4)

*CI* confidence interval

^a^ Denominator is the number of patients evaluated for response

Six of the prespecified variables were identified as prognostic for OS in univariate Cox regression analysis: ECOG PS, *KRAS*-mutation status, exposure to all standard treatments, WBC, serum LDH, and ALP (Table 4). ECOG PS, *KRAS*-mutation status, WBC, serum LDH, and ALP remained statistically significant in multivariate analysis.

**Table 4:** Univariate Cox regression analyses of overall survival

	*n* (100%)	HR	*p* value
Sex			
Male	92 (68)	Ref.	
Female	44 (32)	0.695	0.122
Age (years)			
<70	104 (77)	Ref.	
>70	32 (24)	0.826	0.438
Tumor topography			
Right colon	25 (19)	Ref.	
Left colon and rectum	110 (82)	0.935	0.802
Resection of primary tumor			
No	46 (34)	Ref.	
Yes	90 (66)	0.781	0.263
Presentation of metastases			
Synchronous	93 (68)	Ref.	
Metachronous	43 (32)	0.653	0.071
Time since diagnosis of first metastasis (months)			
< 18	29 (21)	Ref.	
≥ 18	107 (79)	1.014	0.960
Exposure to all standard treatments			
Yes	94 (69)	Ref.	
No	42 (31)	0.550	0.015
Number of metastatic sites			
≥3	85 (63)	Ref.	
2	36 (27)	1.367	0.194
1	15 (11)	1.071	0.836
Peritoneal metastases			
No	94 (69)	Ref.	
Yes	42 (31)	0.675	0.097
*KRAS*-mutation status			
Wild type	53 (39)	Ref.	
Mutated	83 (61)	1.665	0.024
ECOG performance score			
0	46 (34)	Ref.	
1	78 (57)	2.119	0.002
2	12 (9)	2.101	0.089
White blood cell count (×10^9^/l)			
<8.0	66 (49)	Ref.	
≥8.0	70 (52)	1.724	0.010
Serum lactate dehydrogenase (U/l)			
<350	67 (49)	Ref.	
≥350	69 (51)	2.052	0.001
Alkaline phosphatase (U/l)			
<200	76 (56)	Ref.	0.001
≥200	59 (44)	2.087	

*HR* hazard ratio, *ECOG* Eastern Cooperative Oncology Group

## Discussion

We assessed the feasibility and effectiveness of trifluridine/tipiracil in refractory mCRC patients who were treated in a national compassionate use program. Our data indicate that treatment with trifluridine/tipiracil is feasible and safe in daily clinical practice.

Trifluridine/tipiracil was generally well tolerated, with neutropenia as the most frequently observed grade ≥3 adverse event. Three patients were hospitalized for febrile neutropenia. The incidence of nonhematological grade ≥3 toxicities was low, which reflects the low number of patients who discontinued treatment for adverse events. No major new safety concerns were observed in this non-trial population. Although the incidence of any grade toxicities was somewhat lower in our predominantly Caucasian population compared to the global RECOURSE study population, the overall safety profile was comparable, suggesting that there are no ethnic differences in the tolerability of trifluridine/tipiracil. Similar findings have been reported in a subgroup analysis of Spanish RECOURSE study patients [5], in contrast to the safety profile of fluoropyrimidines, where regional disparities have led to different dosing schedules [6, 7].

The effectiveness of trifluridine/tipiracil in our patients treated outside the context of a trial appeared to be inferior to the outcomes in the RECOURSE study. Several comments can be made on this issue.

There are multiple differences between the baseline characteristics of our population and that of the RECOURSE study population. Our population had a worse overall health status, with 57% and 9% having an ECOG PS of 1 or 2, respectively, compared to 44% and 0% in the RECOURSE study population, respectively. Although the scoring of performance status by physicians may not be optimal [8], it is an established prognostic factor in mCRC and in refractory mCRC in particular [9, 10] and was identified as a prognostic parameter in the RECOURSE study. This aspect is reflected in the poor outcomes that we observed in patients with ECOG PS 2. Our data confirm the worse outcomes of trifluridine/tipiracil in patients with *KRAS*-mutated tumors compared to *KRAS*-wild-type tumors, and our patients had a higher incidence of *KRAS*-mutated tumors compared to the RECOURSE patients (61% versus 51%, respectively).

On the other hand, fewer patients in our population were exposed to all available standard therapies before trifluridine/tipiracil treatment, which in this subgroup was associated with a better outcome, suggesting that trifluridine/tipiracil may have better efficacy in earlier lines of treatment, as is currently being investigated. Three phase 2 studies in mCRC patients are ongoing, investigating trifluridine/tipiracil in combination with bevacizumab as maintenance therapy following fluoropyrimidine-based induction therapy (ALEXANDRIA), studying trifluridine/tipiracil in combination with nivolumab in patients with refractory micro-satellite stable mCRC, and comparing trifluridine/tipiracil with capecitabine, both with the addition of bevacizumab, as first-line treatment in patients who are not eligible for combination chemotherapy (TASCO1), respectively (ClinicalTrials.gov identifiers: NCT02654639, NCT02860546, and NCT02743221).

Other factors that may influence a comparison between our efficacy results and those of the RECOURSE study are discrepancies in survival time calculations (time from the start of treatment versus time from randomization, respectively, until date of event), analyzed population (only patients who received at least one dose of trifluridine/tipiracil were included in our analyses), and disease evaluation. Patients in the RECOURSE study were required to have a disease evaluation at least every two cycles, whereas a substantial number of patients in the compassionate use program had their first disease evaluation after three cycles (i.e., 12 weeks). This difference may have distorted the median PFS in our population positively while affecting the DCR negatively.

The superior efficacy of trifluridine/tipiracil compared to placebo in the RECOURSE study was observed in all prespecified subgroups [1]. However, the diverging Kaplan–Meier curves for PFS at the time of the first disease evaluation suggest that only a subgroup of patients may benefit from trifluridine/tipiracil treatment. Kasi et al. and Hamauchi et al. demonstrated that the occurrence of neutropenia was associated with better prognosis and postulated that dose escalation of trifluridine/tipiracil in patients without neutropenia should be further investigated to improve treatment outcomes in these patients [11, 12]. Moreover, potential predictive genetic biomarkers for trifluridine/tipiracil therapy have been identified [13].

In patients with limited life expectancy, the use of potentially harmful end-of-life treatments should be questioned [14]. A proper clinical selection may be helpful to reduce the number of patients who will be unnecessarily exposed to toxicity and to increase cost-effectiveness. Although numerous prognostic parameters have been identified in newly diagnosed mCRC patients [15], limited data are available in heavily pretreated mCRC patients. ECOG PS, elapsed time since diagnosis of first metastasis, and number of metastatic sites were identified as prognostic variables in the RECOURSE study [1]. Pietrantonio et al. developed a predictive nomogram for the 12-week death probability in refractory mCRC patients, with the use of four easy-to-collect variables including ECOG PS, serum LDH, resection of primary tumor, and presence of peritoneal metastases, that may improve the selection of patients for later-line therapies [10]. Our relatively small cohort suggested that poor ECOG PS, a *KRAS*-mutated tumor, and elevated levels of serum LDH, WBC, and ALP are associated with poorer survival outcomes upon trifluridine/tipiracil treatment. These variables may help clinicians to estimate the prognosis in these patients, but further research to validate these findings is needed. Tumor sidedness was not prognostic, which confirms the lack of prognostic value of this parameter in a refractory setting [16].

In conclusion, our data show that treatment with trifluridine/tipiracil in daily clinical practice is feasible. The drug was generally well tolerated, with a safety profile comparable to that of the global RECOURSE study population. Our data argue against the use of trifluridine/tipiracil in refractory mCRC patients with poor performance status, although the outcomes in less heavily pretreated patients support future studies in earlier lines of treatment. Further research to identify predictive factors to reduce the number of patients who would be unnecessarily exposed to toxicity and to increase cost-effectiveness is warranted.

## References

[1] Mayer RJ, Van Cutsem E, Falcone A (2015). Randomized trial of TAS-102 for refractory metastatic colorectal cancer. N Engl J Med.

[2] Tanaka N, Sakamoto K, Okabe H (2014). Repeated oral dosing of TAS-102 confers high trifluridine incorporation into DNA and sustained antitumor activity in mouse models. Oncol Rep.

[3] Emura T, Suzuki N, Fujioka A (2005). Potentiation of the antitumor activity of alpha, alpha, alpha-trifluorothymidine by the co-administration of an inhibitor of thymidine phosphorylase at a suitable molar ratio in vivo. Int J Oncol.

[4] Fukushima M, Suzuki N, Emura T (2000). Structure and activity of specific inhibitors of thymidine phosphorylase to potentiate the function of antitumor 2′-deoxyribonucleosides. Biochem Pharmacol.

[5] Longo-Muñoz F, Argiles G, Tabernero J (2017). Efficacy of trifluridine and tipiracil (TAS-102) versus placebo, with supportive care, in a randomized, controlled trial of patients with metastatic colorectal cancer from Spain: results of a subgroup analysis of the phase 3 RECOURSE trial. Clin Transl Oncol.

[6] Haller DG, Cassidy J, Clarke SJ (2008). Potential regional differences for the tolerability profiles of fluoropyrimidines. J Clin Oncol.

[7] Kwakman JJ, Punt CJ (2016). Oral drugs in the treatment of metastatic colorectal cancer. Expert Opin Pharmacother.

[8] Mol L, Ottevanger PB, Koopman M (2016). The prognostic value of WHO performance status in relation to quality of life in advanced colorectal cancer patients. Eur J Cancer.

[9] Köhne CH, Cunningham D, Di Costanzo F (2002). Clinical determinants of survival in patients with 5-fluorouracil-based treatment for metastatic colorectal cancer: results of a multivariate analysis of 3825 patients. Ann Oncol.

[10] Pietrantonio F, Miceli R, Rimassa L (2017). Estimating 12-week death probability in patients with refractory metastatic colorectal cancer: the Colon Life nomogram. Ann Oncol.

[11] Kasi PM, Kotani D, Cecchini M (2016). Chemotherapy induced neutropenia at 1-month mark is a predictor of overall survival in patients receiving TAS-102 for refractory metastatic colorectal cancer: a cohort study. BMC Cancer.

[12] Hamauchi S, Yamazaki K, Masuishi T (2017). Neutropenia as a predictive factor in metastatic colorectal cancer treated with TAS-102. Clin Colorectal Cancer.

[13] Suenaga M, Schirripa M, Cao S (2017). Genetic variants of DNA repair-related genes predict efficacy of TAS-102 in patients with refractory metastatic colorectal cancer. Ann Oncol.

[14] Prigerson HG, Bao Y, Shah MA (2015). Chemotherapy use, performance status, and quality of life at the end of life. JAMA Oncol.

[15] Punt CJ, Koopman M, Vermeulen L (2017). From tumour heterogeneity to advances in precision treatment of colorectal cancer. Nat Rev Clin Oncol.

[16] Brulé SY, Jonker DJ, Karapetis CS (2015). Location of colon cancer (right-sided versus left-sided) as a prognostic factor and a predictor of benefit from cetuximab in NCIC CO.17. Eur J Cancer.

